# Rapid detection of ricin at trace levels in complex matrices by asialofetuin-coated beads and bottom-up proteomics using high-resolution mass spectrometry

**DOI:** 10.1007/s00216-024-05452-0

**Published:** 2024-07-24

**Authors:** Paloma Piquet, Justyna Saadi, François Fenaille, Suzanne R. Kalb, François Becher

**Affiliations:** 1 Département Médicaments Et Technologies Pour La Santé (DMTS), INRAE, CEA, Université Paris-Saclay, 91191 Gif-Sur-Yvette, France; 2 Division of Laboratory Sciences, Centers for Disease Control and Prevention, National Center for Environmental Health, Atlanta, GA 30341 USA

**Keywords:** Asialofetuin, Ricin, High-resolution mass spectrometry, Enrichment, Sample preparation, Toxin

## Abstract

Ricin is a toxic protein regarded as a potential chemical weapon for bioterrorism or criminal use. In the event of a ricin incident, rapid analytical methods are essential for ricin confirmation in a diversity of matrices, from environmental to human or food samples. Mass spectrometry–based methods provide specific toxin identification but require prior enrichment by antibodies to reach trace-level detection in matrices. Here, we describe a novel assay using the glycoprotein asialofetuin as an alternative to antibodies for ricin enrichment, combined with the specific detection of signature peptides by high-resolution mass spectrometry. Additionally, optimizations made to the assay reduced the sample preparation time from 5 h to 80 min only. Method evaluation confirmed the detection of ricin at trace levels over a wide range of pH and in protein-rich samples, illustrating challenging matrices. This new method constitutes a relevant antibody-free solution for the fast and specific mass spectrometry detection of ricin in the situation of a suspected toxin incident, complementary to active ricin determination by adenine release assays.

**Graphical Abstract:**

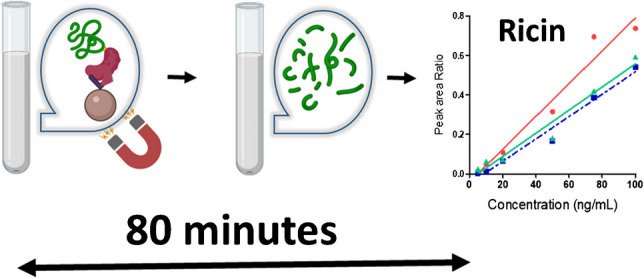

**Supplementary Information:**

The online version contains supplementary material available at 10.1007/s00216-024-05452-0.

## Introduction

Ricin is a glycosylated protein of ∼62 kDa found in abundance in the seeds of the castor bean plant *Ricinus communis* [1]. The ricin toxin is composed of two disulfide-linked chains. The B-chain (RTB) binds to terminal galactosyl residues on cell surface glycoproteins and glycolipids, inducing ricin internalization; the A-chain (RTA) stops protein synthesis by depurination of the 28S rRNA in the 60S ribosome subunit [2]. Two isoforms of ricin have been reported, ricin D and ricin E, with identical RTA and a 15% difference in amino acid composition for RTB [3, 4], along with the highly homologous but less toxic protein *Ricinus communis* agglutinin also present in seeds [5].

Ricin is classified as a prohibited substance by the Chemical Weapons Convention (CWC, schedule 1 compound) and the Biological Weapons Convention (BWC) because of its wide availability, relative ease of preparation, and high toxicity in humans [6]. In the situation of a potential ricin incident, unambiguous identification of ricin requires a panel of methods combining complementary analytical approaches and selectivity. Also, time to response is crucial to confirm as fast as possible the presence of ricin in a suspect sample, which could be of environmental, food, or human source [7]. For these reasons, new and rapid methods for ricin detection must be developed and applicable to a variety of complex matrices.

Many ricin detection methods are based on antibodies. Selective binding to high-affinity antibodies supports the detection of ricin by ELISA or lateral flow assay formats [8]. Immuno-enrichment antibodies are combined with a specific bottom-up mass spectrometry detection of signature peptides released from the toxin in complex samples [9–14]. However, antibodies against ricin are frequently proprietary from a laboratory, affecting exchanges or transfers of the methods. Also, antibodies with narrow specificity may recognize exclusively one isoform of ricin, resulting in a partial detection [15].

As an alternative to antibodies, the ricin B-chain’s ability to bind galactosyl moieties has been exploited for affinity purification from ricin beans, beverages, or powders [16–19]. In these reports, galactose or lactose was used as ligand, coupled to chromatographic materials [16] or magnetic beads [17]. A glycosylated protein would better mimic the binding of ricin to the cellular membrane and would be easier to couple to the surface of magnetic beads using similar conditions to antibodies [20]. The commercially available glycoprotein asialofetuin was recently shown to bind ricin B-chain efficiently and allowed the functional detection of ricin by adenine release assay on a MALDI-TOF instrument [21]. Affinity enrichment by asialofetuin-coated beads was combined here with targeted high-resolution mass spectrometry detection of ricin peptides. The new method was able to detect ricin with high specificity and similar sensitivity to the adenine release assay in human serum and soup, constituting a complementary approach for confirmation of ricin contamination in complex samples. In addition, acceleration of the protocol was investigated resulting in the detection of ricin at trace levels in 80 min only.

## Experimental

### Chemicals and reagents

Phosphate-buffered saline (PBS), phosphate-buffered saline Tween 1 × (PBST 1 ×), sodium phosphate, ammonium bicarbonate, bovine serum albumin (BSA), dithiothreitol (DTT), iodoacetamide (IAA), formic acid (LC–MS grade), hydrochloric acid (HCl), sodium hydroxide, disodium hydrogen phosphate, sodium chloride, deoxycholic acid, and asialofetuin type I were from Sigma-Aldrich-Merck (Saint Quentin Fallavier, France). Water (LC–MS grade) and acetonitrile (LC–MS grade) were obtained from Honeywell/Riedel–de Haen (Seelze, Germany) and Biosolve Chimie (Dieuze, France), respectively. Sequencing-grade modified trypsin was obtained from Promega Corporation (Charbonnieres-les-Bains, France). Dynabeads M-280 tosylactivated magnetic beads were obtained from Invitrogen (Life Technologies, Oslo, Norway). Labelled peptides for quantification were synthesized in Absolute QUAntitation (AQUA) ultimate quality by ThermoFisher Scientific (Paisley, UK). For all reactions, LoBind Eppendorf tubes (Dutscher, Brumath, France) were used. Ricin isoforms D and E were obtained from Dr Delgado and purified as previously described [15].

### Safety precaution

Due to the high toxicity of ricin, experiments were performed with strict adherence to safety rules for the handling of toxic substances, involving trained personnel only and experimental work under a class II vertical laminar flow cabinet (Thermo Fisher Scientific, Les Ulis, France). Toxin-contaminated solutions and consumables were inactivated overnight with 2 M NaOH.

### Sample preparation for ricin detection

The glycoprotein asialofetuin was coupled to Dynabeads M-280 tosylactivated magnetic beads [10] with a ratio of 350 μL of beads to 200 μL of asialofetuin (1 mg/mL) and incubated overnight in sodium phosphate buffer. Beads were resuspended and kept at 4 °C in PBS with BSA at 0.1%. The mixture of magnetic beads coated with asialofetuin was used for immunocapture, using 40 µL of the bead suspension added to each sample. Incubation and washing steps were performed on a King Fisher Duo Prime robot (Thermo Fisher Scientific, Bremen, Germany). Incubation with the beads was generally done in a total volume of 1 mL after dilution of the sample in PBS Tween 1X containing 0.1% BSA, or otherwise stated in the result section, for 1 h at RT. Successive bead washing steps with PBS-Tween 1X, PBS, and pure water were performed before the final transfer in 40 μL of deoxycholic acid at 1% in 50 mM ammonium bicarbonate. The on-beads digestion was done as follows. The elution strip containing the bead was heated for 15 min at 95 °C for denaturation. After cooling at room temperature, digestion was performed with 2 μg of sequencing-grade modified trypsin (2 μL of 1 μg/μL in H_2_O) for 10 min at 70 °C. After incubation, the digestion was stopped by adding 5 μL of 1 M HCl. The elution strip was centrifuged (3000 rpm, 5 min) and the supernatant was transferred to MS vials in addition to 10 μL of the mixture of labelled peptides. Twenty microliters was injected into the LC–MS system.

### Liquid chromatography–high-resolution mass spectrometry

LC–MS/MS data were acquired using a Dionex Ultimate 3000 system coupled to a Q-Exactive mass spectrometer (Thermo Fisher Scientific, Bremen, Germany) using an Aeris peptide XBC18 reverse phase column (150 mm × 2.1 mm; 1.7 μm; 100 Å; Phenomenex, Le Pecq, France), in chromatography and MS conditions similar to those reported by our group [22]. The column oven temperature was set to 60 °C. Mobile phases consisting of LC–MS-grade water with 0.1% formic acid (phase A) and acetonitrile with 0.1% formic acid (phase B) were delivered at a flow rate of 0.5 mL/min. A linear gradient was applied for a total run time of 13 min [22]. Eluted peptides were introduced into the Q-Exactive instrument, operating in positive electrospray ionization (ESI) mode under time-scheduled sequential parallel reaction monitoring (PRM) acquisition. Instrument parameters of the atmospheric pressure source were as follows: sheath gas flow rate 70 arbitrary units, spray voltage 4 kV, capillary temperature 280 °C. Precursor ions from native and labelled peptides were selected in the quadrupole (see Electronic Supplementary Material Table S1, Supporting Information) with an isolation mass window of 1.5 Da. They were fragmented in the HCD cell and fragmented using nitrogen as collision gas and the given normalized collision energy (see Electronic Supplementary Material Table S1**)**. All fragment ions were transferred to the Orbitrap. Resolution was set to 35,000 at *m/z* 200 (full width at half-maximum), automatic gain control to 1e^6^, and maximum injection time to 125 ms. Xcalibur 2.2 software (Thermo Fisher Scientific, Bremen, Germany) was used for instrument control and processing of the data files. Peak area was determined as the sum of the signal from the 3 most abundant fragment ions. The peptide map of ricin was obtained by data-dependent acquisition as previously reported [10].

### Matrices

The pea soup (GreenShoot, Velouté de Petits Pois) was purchased in a local supermarket. Human serum was obtained from Merck (Darmstadt, Germany).

## Results and discussion

### Ricin enrichment by asialofetuin-coated beads

Detection of ricin at trace level in complex samples requires purification of the toxin before mass spectrometry analysis by immunomagnetic separation [23–27]. As an alternative to antibodies, the commercially available glycoprotein asialofetuin binds ricin B-chain efficiently [28–30], mimicking the binding to the cellular membrane and allowing the detection of ricin activity by adenine release assay on a MALDI-TOF instrument [21]. Here, we evaluated the enrichment using asialofetuin for the detection of ricin in complex samples by signature peptides released from the toxin and high-resolution mass spectrometry in PRM mode. In this mode, all fragment ions from the targeted ricin peptides are simultaneously detected, at high resolution and high mass accuracy for specific mass spectrometry detection and noise reduction [10].

**Fig. 1:**
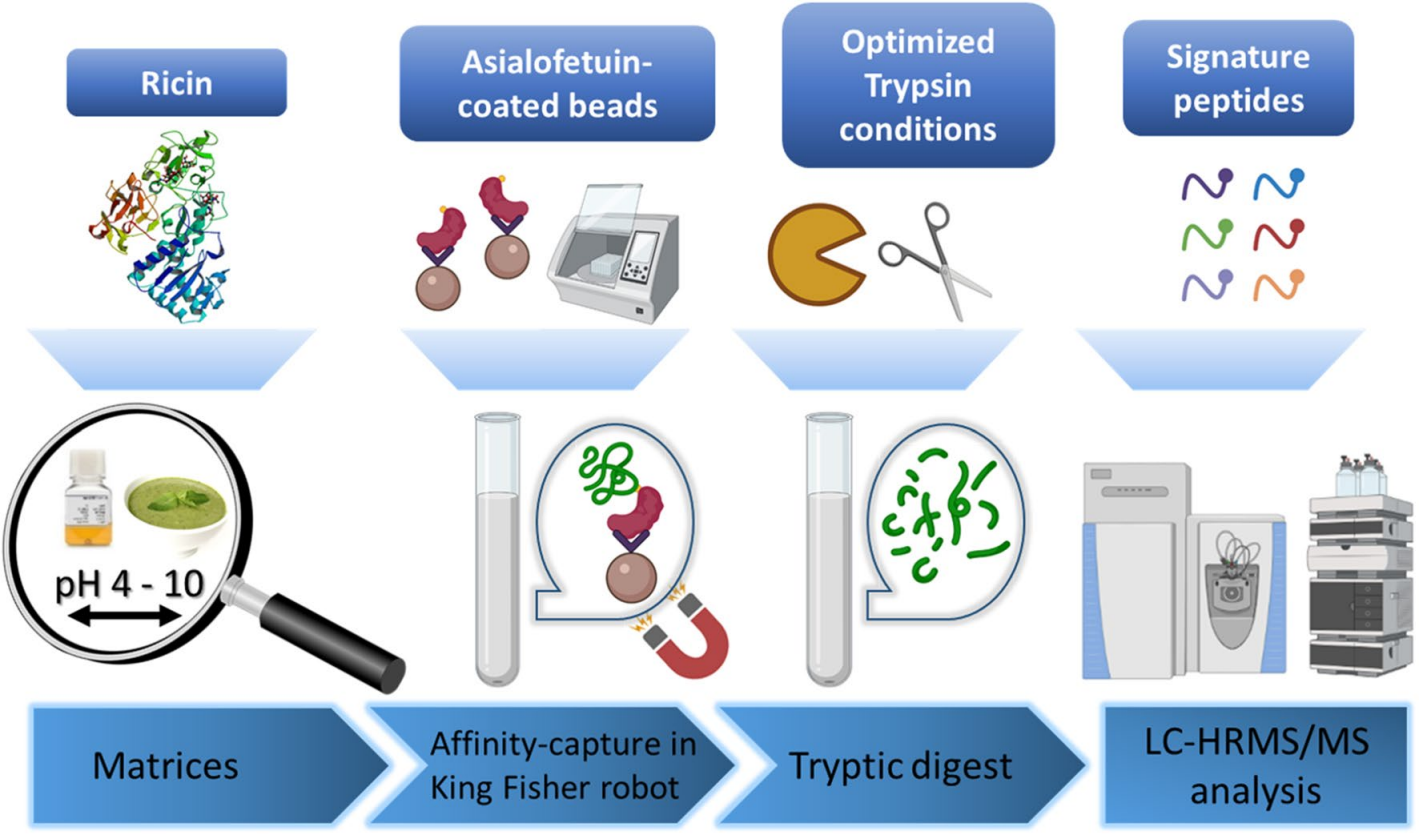
Detection of ricin by combining asialofetuin-coated beads and high-resolution mass spectrometry

Capture conditions of ricin were established according to the on-beads immuno-enrichment protocol reported previously by our group [22], substituting ricin-specific antibodies to asialofetuin, while maintaining similar magnetic bead volume and ligand amount. The glycoprotein has 12 terminal galactose residues per molecule, resulting in a high affinity for ricin [31, 32]. The protocol is illustrated in Fig. 1.

**Fig. 2:**
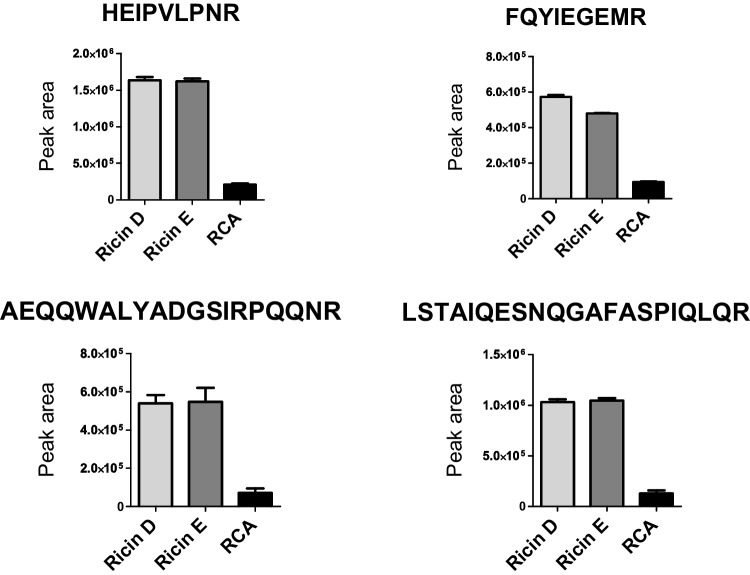
Detection of ricin isoforms D and E and agglutinin by mass spectrometry after capture by asialofetuin. Ricin D, ricin E, and agglutinin were spiked at 100 ng/mL in BSA buffer, enriched by asialofetuin-coated beads, and trypsin-digested. Peptides common to ricin D and E and agglutinin were measured by PRM mass spectrometry, *n* = 3. Peak area (± standard error of the mean, SD) is illustrated

The ability of asialofetuin beads to bind the two different isoforms of ricin, i.e., ricin D and ricin E, was evaluated. Capture of the less toxic *Ricinus communis* agglutinin (RCA) was also tested, while discrimination of agglutinin from ricin is usually possible by mass spectrometry [33]. Four peptides shared between the two ricin isoforms and agglutinin were monitored by high-resolution mass spectrometry and used to assess the binding efficiency of the purified ricin isoforms or agglutinin spiked separately at an equivalent amount of 100 ng/mL (Fig. 2). The signal of the four peptides was around a factor 8 higher compared to agglutinin when released either from ricin D or E, showing a preferential capture of ricin by the asialofetuin-coated beads. The difference could be explained by distinct glycoforms of ricin and agglutinin, favoring the capture of the ricin toxin [34, 35]. The capture recovery was measured by spiking ricin D before and after enrichment by asialobeads and determined at 71%, similar to data reported for immuno-enrichment protocols [10, 22]. The protocol was therefore found efficient and able to bind both isoforms of ricin.

### Rapid detection of ricin with asialofetuin-coated beads and mass spectrometry

**Fig. 3:**
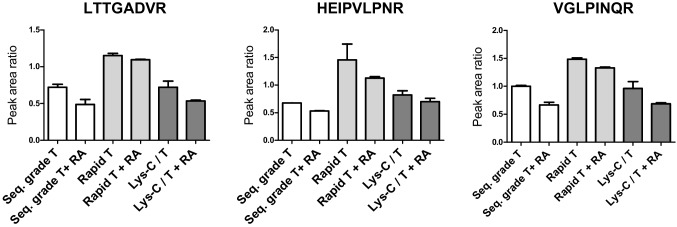
Sequencing-grade modified trypsin, Rapid-digestion trypsin, and Lys-C-trypsin mix with or without reduction/alkylation (RA) for ricin on-beads digestion. Incubation time was 1 h at 70 °C and trypsin amount was 1 µg/mL for the Rapid trypsin and the mix Lys-C/trypsin. Incubation time was 2 h at 37 °C and trypsin amount was 0.1 µg/mL for the sequencing-grade trypsin. Peptide PRM signal (peak area ratio with labelled peptide, ± SD) was determined after enrichment by asialofetuin-coated beads and digestion of ricin spiked at 50 ng/mL in BSA buffer, *n* = 2

To limit the time-to-response for ricin detection, we wished to establish accelerated conditions for the new assay based on asialofetuin beads and mass spectrometry. Sample preparation was the focus of the optimizations, i.e., affinity purification with the asialofetuin beads, on-beads toxin denaturation, and finally digestion by trypsin which is the most time-consuming step of the analytical protocol. In the initial protocol (Fig. 1), the toxin binds to the asialofetuin-coated beads, and the peptides are directly released from the beads following the addition of denaturing agents and trypsin. Denaturation and on-beads digestion required more than 4 h, comprising 15-min denaturation with RapiGest SF surfactant, 60-min reduction/alkylation of cysteine residues, 2-h digestion with sequencing-grade modified trypsin, and 45-min lysis of RapiGest SF surfactant. The impact of protocol optimizations was evaluated by the signal of the 3 best ricin peptides usually monitored by mass spectrometry, normalized to the corresponding labelled peptide (see Electronic Supplementary Material Table S1) [9, 10, 33]. First, we compared the peptide signal using either our protocol with the sequencing-grade trypsin [22] or the Rapid trypsin from Promega which is reported for shortened digestion time [36]. Incubation with the Rapid trypsin was performed as recommended [36], during 1 h at 70 °C and a high excess amount of enzyme (1 µg/µL) (Fig. 3). We also tested a mix of LysC and Rapid trypsin to promote the release of peptides, in the same conditions [37]. For the three peptides, the best signal was observed with the Rapid trypsin condition showing a twofold improvement compared to the sequencing-grade trypsin or LysC-trypsin conditions (Fig. 3). When using the same incubation conditions with the sequencing-grade trypsin, i.e., incubation at 70 °C and 1 µg/mL trypsin, similar peptide signals to the Rapid trypsin were obtained (see Electronic Supplementary Material Fig. S1). Additionally, we assessed the benefit of reduction and alkylation before digestion in all digestion conditions. We observed a slight loss of signal following the reduction and alkylation steps (Fig. 3), probably due to the dilution of the digests, suggesting that the heating step and the addition of a detergent are sufficient for the denaturation of ricin and the release of the 3 peptides.

RapiGest SF, an acid-labile surfactant, was reported for efficient in-solution or on-beads denaturation before the digestion of ricin [10, 38]. However, RapiGest SF requires 45-min inactivation by acid at 37 °C and 15-min centrifugation. Alternatively, a 1% deoxycholic acid (DOC) solution was reported for the efficient denaturation of proteins [39] and requires only acidification for elimination. A similar or better signal of the three peptides was obtained using DOC at 1% (see Electronic Supplementary Material Fig. S2), compared to the condition with RapiGest SF 0.05%, indicating similar ricin denaturation efficiency. After optimization of the digestion conditions, we selected DOC at 1% for denaturation together with the Sequencing-grade trypsin at 1 µg/µL incubated at 70 °C, without reduction/alkylation.

**Fig. 4:**
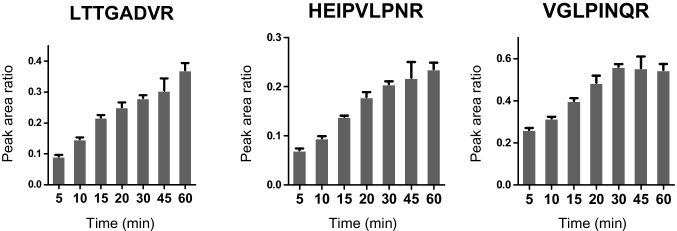
On-beads digestion kinetic of ricin in optimized conditions. Incubation time was 5 min to 1 h at 70 °C and trypsin amount was 1 µg/mL using the sequencing-grade modified trypsin. Peptide PRM signal (peak area ratio with labelled peptide, ± SD) was determined after enrichment by asialofetuin-coated beads and digestion of ricin spiked at 20 ng/mL in BSA buffer, *n* = 2

We tested shortened incubation times with trypsin, ranging between 5 min and 1 h. A gradual increase in signal was observed for all peptides over time. The maximum signal was reached between 30 and 60 min of incubation depending on the peptide (Fig. 4). At 10 min, the signal was around two to threefold lower for the three peptides, compared to the maximum as shown in Fig. 4. We selected the 10-min incubation time with trypsin considering most important the drastic reduction in time.

We also applied different binding times of the toxin with the asialofetuin-coated beads, varying between 15 and 120 min. A similar signal of the 3 peptides was observed, with a slight tendency to decrease at 15 min. An incubation time of 30 min was selected for the method (see Electronic Supplementary Material Fig. S3).

In the end, the total sample preparation time was 80 min, including 30-min incubation with asialofetuin-coated beads, 20-min beads washing, 15-min denaturation with DOC, and 10-min incubation with trypsin.

### Evaluation of the rapid protocol for ricin detection in matrices

The sensitivity and repeatability of the new assay were determined in typical complex matrices. We selected acidic or basic buffers and protein-rich matrices to challenge the binding of ricin to asialofetuin. For the method evaluation, we kept the three good responder peptides for MRM or PRM methods, LTTGADVR, VGLPINQR, and HEIPVLPNR (Table S1) [9, 10, 27]. The use of similar peptides to previous mass spectrometry methods facilitates the comparative evaluation of the new assay. Also, monitoring peptides from the A-chain allows the detection of the entire toxin, since asialofetuin binds ricin through the B-chain. In parallel, we determined the ability of the accelerated asialofetuin beads protocol to detect other peptides from the two chains of ricin. In this aim, a peptide map was generated at a low ricin concentration by substituting PRM by data-dependant acquisition. Seven additional peptides were identified along the sequence, including 4 from the A-chain and 3 from the B-chain. Among them, two were specific to ricin D and/or ricin E and not present in agglutinin, allowing confident identification of the toxin (Table 1).

**Table 1:** Additional peptides detected at 50 ng/mL by data-dependent acquisition (DDA). The Uniprot sequence entry P02879 was considered. Only peptides not containing cysteine were identified since the reduction and alkylation steps were removed from the protocol

Peptide sequence	Position	Chain	Origin	Theo. MH + (Da)	Xcorr
LEQLAGNLR	161–169	A	Ricin D, ricin E	1013.5738	1.58
FQYIEGEMR	216–224	A	Ricin D, ricin E, RCA	1172.5405	1.55
SAPDPSVITLENSWGR	233–248	A	Ricin D, ricin E, RCA	1728.8551	0.93
LSTAIQESNQGAFASPIQLQR	249–269	A	Ricin D, ricin E, RCA	2259.1728	3.58
SNTDANQLWTLK	355–366	B	Ricin D, ricin E	1390.6961	1.08
AEQQWALYADGSIRPQQNR	483–501	B	Ricin D, ricin E, RCA	2231.0952	1.51
NDGTILNLYSGLVLDVR	534–550	B	Ricin D	1862.0018	1.73

**Fig. 5:**
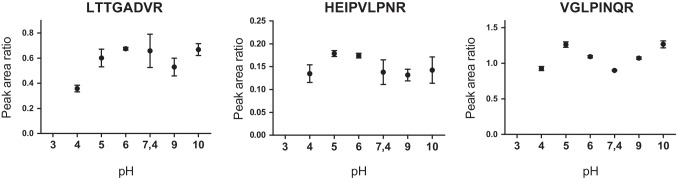
Impact of the binding buffer pH on the signal of ricin peptides. Peptide PRM signal (peak area ratio with labelled peptide, ± SD) was determined after enrichment by asialofetuin-coated beads and digestion of ricin spiked at 50 ng/mL in phosphate buffers adjusted at different pH, *n* = 2

The impact of pH on the capture by asialofetuin-coated beads was evaluated by testing acidic and basic binding buffers (Fig. 5). In the situation of a potential incident, the determination of ricin within various unknown sample matrices might be requested. Dilution by buffers before capture may not adjust the pH perfectly depending on the nature of the original matrix. The impact of the pH during binding should be considered because of the potential denaturation of the ligand or the toxin. Here, the pH of binding buffer ranging from 3 to 10 was applied during ricin capture. No signal was detected at pH 3, then the three peptides were well detected in the range pH 4 to pH 10 (Fig. 5). The results indicate the compatibility of the affinity capture by asialofetuin with minimal influence of pH on the sugar-ricin interactions in the range pH 4 to pH 10.

**Fig. 6:**
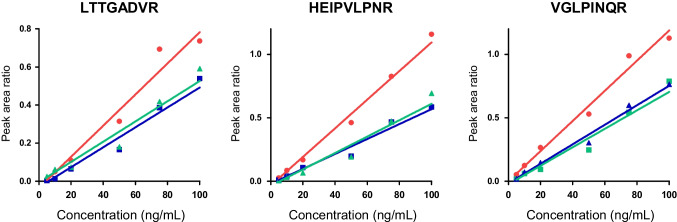
Linearity of the accelerated protocol in buffer (red), human serum (blue), and soup (green); signal of the three peptides is illustrated

The method was evaluated in human serum and pea soup, two difficult matrices containing high-protein backgrounds, in parallel to phosphate buffer containing only albumin. LOD was determined by serial dilution of ricin in the matrices and was found between 1 and 5 ng/mL depending on the peptides and the matrix (Table 2). The lowest ricin concentration where a positive signal was detected for the three peptides was 2 ng/mL in buffer and 5 ng/mL in soup or human serum. The reduced slope of the curves confirmed a slight matrix effect in the two difficult matrices compared to the buffer (Fig. 6). Overall, the detection limits determined here appear similar to antibody-enrichment-based assays operated on similar mass spectrometry settings [9, 10]. We observed good repeatability overall with CV below 20% in buffer and soup with peptides LTTGADVR and VGLPINQR (Table 2). Higher variability, in the range 26.8–40.0% (*n* = 5), was detected in the human serum matrix. Acceleration of the protocol, notably the short digestion time by trypsin, together with the higher total concentration of serum protein may explain the result. In the situation of a positive sample detected by the accelerated protocol, precise ricin measurement with quantitative immunological or MS assays [8, 40] would be required.

**Table 2:** CV%, LOD, and linearity of the accelerated protocol for ricin detection in buffer, human serum, and pea soup for 3 ricin peptides. The CV was calculated with independent QCs spiked at 20 ng/mL (*n* = 5 QCs)

	LTTGADVR			HEIPVLPNR			VGLPINQR		
	Buffer	Serum	Soup	Buffer	Serum	Soup	Buffer	Serum	Soup
LOD (ng/mL)	2	5	2	2	2	5	1	2	2
CV%	9	39.8	14.7	23.7	32.5	4.5	9.5	26.8	12.7
*r*^2^	0.975	0.970	0.952	0.992	0.975	0.958	0.988	0.988	0.967

## Conclusion

We applied a simple ricin extraction strategy, relying on the affinity of ricin B-chain for the commercially available glycoprotein asialofetuin coated on magnetic beads. Detection in the low ng/mL range was achieved allowing the detection of ricin at trace levels in two challenging matrices. Furthermore, the assay was optimized for accelerated conditions, resulting in a total time for sample preparation of 80 min only. The LOD was found similar to antibody-capture mass spectrometry assays, while most of those assays required a day or more for capture and digestion. We also showed the efficiency of asialofetuin beads for affinity capture of ricin at trace levels over a wide range of pH. The combination reported here of asialofetuin-beads extraction with high-resolution mass spectrometry provides a complementary approach to the detection of active ricin by adenine release assay [21]. In the event of a bioterrorism attack, the method can rapidly confirm the detection results obtained with field detection assays by the unambiguous detection of signature peptides, and discriminate further ricin with the less toxic agglutinin protein through peptides specific to ricin. In case of positive results, additional measurements by quantitative assays would be required to determine precisely the amount of ricin in the suspect samples. Alternatively, the new method could be evaluated for quantification of ricin through dedicated validation experiments including determination of the limit of quantification, matrix effects, reproducibility, and measurement uncertainty. Additionally, the method could be easily transferred and implemented in regulatory expert laboratories, or extended to the detection of signature peptides from other RIP II toxins, including the abrin toxin.

## Supplementary Information

Below is the link to the electronic supplementary material.

Supplementary file1 (PDF 94 KB)
