# Supplementary material for: Rapid detection of ricin at trace levels in complex matrices by asialofetuin-coated beads and bottom-up proteomics using high-resolution mass spectrometry
Source: Anal Bioanal Chem. 2024 Jul 24;416(23):5145–53. doi: 10.1007/s00216-024-05452-0 (PMC11377644; doi:10.1007/s00216-024-05452-0)
Supplement: Supplementary file 1 — Supplementary file1 (PDF 94 KB) [file 216_2024_5452_MOESM1_ESM.pdf]

SUPPLEMENTARY DATA

Peptides	m/z of precursor ion	Retention time (min)	Normalized collision energy	Charge
**LTTGADVR**	417.2	2.5	15	2
**LTTGADVR[$^{13}C_6$,$^{15}N_4$]**	421.9	2.5	15	2
**HEIPVLPNR**	538.2	4.9	25	2
**HEIPVLPNR[$^{13}C_6$,$^{15}N_4$]**	543.1	4.9	25	2
**VGLPINQR**	449.2	5.1	20	2
**VGLPINQR[$^{13}C_6$,$^{15}N_4$]**	454.0	5.1	20	2

Table S1 : Ricin peptides targeted by PRM for method optimization and evaluation, including labelled peptide versions. Amino acid sequences, retention time, and PRM parameters are indicated

For Peer Review

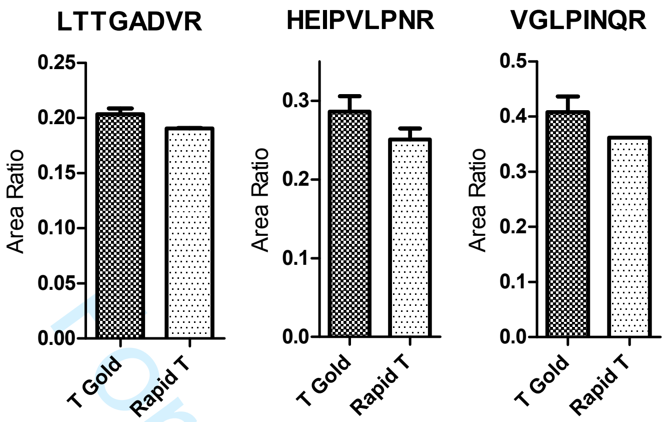

Fig.S1 : Evaluation of Sequencing-grade Trypsin and Rapid trypsin in similar conditions. Peptide PRM signal was determined after asialofetuin enrichment and digestion of ricin spiked at 20 ng/mL in BSA-buffers, incubation with trypsin was done during 1h at 70°C and enzyme concentration at 1 µg/µL.

For Peer Review

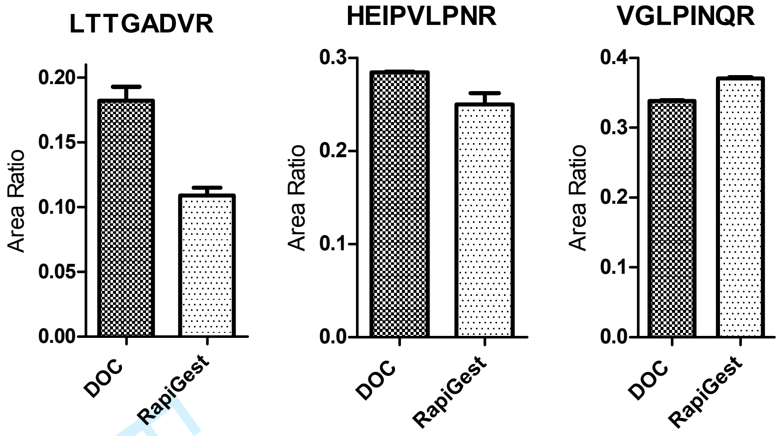

Fig.S2 : Evaluation of 1% DOC and 0.05% RapiGest SF for denaturation of ricin. Peptide PRM signal was determined after asialofetuin enrichment and digestion of ricin spiked at 20 ng/mL in BSA-buffers, incubation with trypsin for 1h at 70°C and enzyme at 1 µg/µL.

For Peer Review

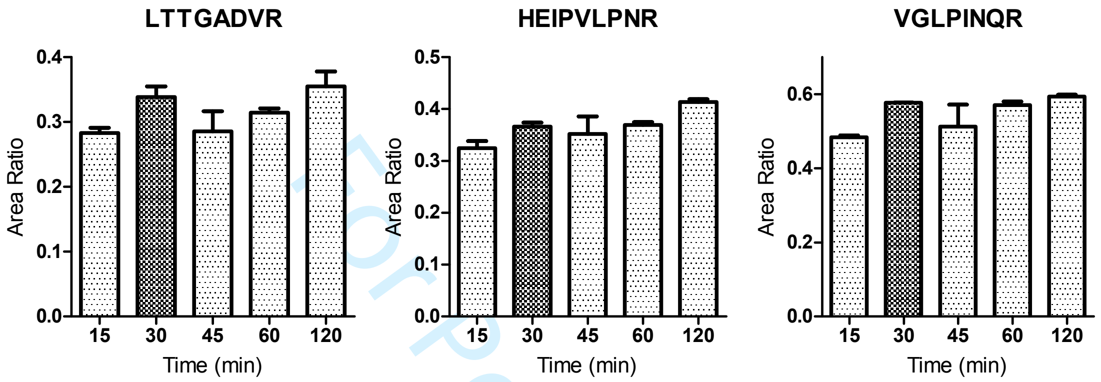

Fig.S3 : Binding kinetic of ricin to asialofetuin-coated beads. Peptide PRM signal was determined after asialofetuin enrichment and digestion of ricin spiked at 20 ng/mL in BSA-buffers, incubation with trypsin for 1h at 70°C and enzyme at 1 µg/µL.

For Peer Review
